# Highlights from the ISCB Student Council Symposia in 2016

**DOI:** 10.12688/f1000research.10389.1

**Published:** 2016-12-15

**Authors:** Bart Cuypers, Annika Jacobsen, Ben Siranosian, Kevin Schwahn, Ashley Mae Conard, Nanne Aben, Mehedi Hassan, Nazeefa Fatima, Susanne M.A. Hermans, Melissa Woghiren, Pieter Meysman, Farzana Rahman, Anupama Jigisha

**Affiliations:** 1 Molecular Parasitology Unit, Department of Biomedical Sciences, Institute of Tropical Medicine, Antwerp, Belgium; 2 Advanced Database Research and Modeling group (ADReM), Department of Mathematics and Computer Science, University of Antwerp, Antwerp, Belgium; 3 Centre for Integrative Bioinformatics (IBIVU), VU University Amsterdam, Amsterdam, Netherlands; 4 Broad Institute, MIT and Harvard, Cambridge, USA; 5 Max Planck Institute of Molecular Plant Physiology, Postdam, Germany; 6 Center for Computational Molecular Biology, Brown University, Providence, USA; 7 Computational Cancer Biology, The Netherlands Cancer Institute, Amsterdam, Netherlands; 8 Genomics and Computational Biology Research Group, Faculty of Computing, Engineering and Science, University of South Wales, Treforest, UK; 9 Eukaryotic Evolution Research Group, School of Applied Sciences, University of Huddersfield, Huddersfield, UK; 10 Institute for Pharmaceutical and Medicinal Chemistry, Heinrich Heine University Düsseldorf, Düsseldorf, Germany; 11 Department of Computing Science, University of Alberta, Edmonton, Canada; 12 Conway Institute of Biomolecular and Biomedical Research, University College Dublin, Dublin, Ireland

**Keywords:** ISCB Student Council, Student Council Symposium, European Student Council Symposium, Meeting highlights, Networking events, Public engagement, Science communication, Outreach

## Abstract

This editorial provides a brief overview of the 12th International Society for Computational Biology (ISCB) Student Council Symposium and the 4th European Student Council Symposium held in Florida, USA and The Hague, Netherlands, respectively. Further, the role of the ISCB Student Council in promoting education and networking in the field of computational biology is also highlighted.

## Introduction

The Student Council (SC) of the International Society for Computational Biology (ISCB) is an international network of young researchers in computational biology and related fields. It strives to provide a platform that complements formal education by organizing opportunities such as symposiums, internships, soft-skill training workshops, and career events. Through the provision of such events and other networking opportunities, and support to Regional Student Groups (RSG) across the world, the SC aims to promote the development of future researchers in the field.

The symposiums are the most successful SC initiative, catering to students across the world with one annual event (in USA or Europe) and three biennial ones (in Europe, Latin America, and Africa). The Student Council Symposium (SCS) is the flagship event on the SC calendar. It started as a satellite meeting of Intelligent Systems for Molecular Biology (ISMB)/European Conference of Computational Biology (ECCB) in Madrid, Spain in 2005 and has now grown to an annual event that is periodically complemented with the European Student Council Symposium (ESCS), the Student Council Symposium-Africa (SCS-Africa) and the Latin America Student Council Symposium (LA-SCS). The primary goal of these symposiums is to provide young researchers in computational biology with the opportunity to present their work to an international audience, gain insights into the latest topics and methods, educate them with outstanding keynote talks by renowned researchers and offer them the occasion to build a worldwide network within the computational biology community. Whereas 2015 only saw the eleventh SCS in Dublin, Ireland as ISMB/ECCB were jointly organized ^1^, both SCS and ESCS took place in 2016.

This editorial showcases the highlights of the twelfth SCS and fourth ESCS.

## Twelfth Student Council Symposium in Orlando, Florida

The 12 ^th^ edition of SCS was organized to take place in Orlando, Florida on July 8, 2016, directly preceding the 24th conference on ISMB. This year, 45 delegates were welcomed from all over the world, and 15 student talks, 27 poster presentations and two keynote speakers were hosted.

The first keynote speaker was Prof. John Quackenbush, who is an expert in the field of genomics and cancer systems biology. He is the Director of the Center for Cancer Computational Biology at the Dana Farber Cancer Institute and a Professor in the Department of Biostatistics at the Harvard School of Public Health. The theme of his talk was on the use of network analysis tools to link genotype and phenotype. Additionally, he introduced his group’s method to reverse engineer sample specific networks, a concept that was new to most attendees. The second keynote presentation was by Prof. Janet Thornton, who is a world leader in structural bioinformatics. She is currently a group leader at the European Bioinformatics Institute (EBI), of which she was also the Director from 2001–2015. Prof. Thornton played a major role in establishing ELIXIR, and is currently a board member of the organization. She co-chaired ISMB/ECCB in 2014. Her talk focused on how to pursue an academic career, using her own track as example. She conveyed potential difficulties one may face while advising on how to produce excellent scientific research. This was of particular interest for the student audience, most of them being at the start of their scientific career.

The student presentations and posters covered a wide variety of topics in Computational Biology. In contrast to other more specialized meetings, the SCS topics ranged from fundamental biology and development of tools and algorithms to the application of tools to biological problems. The breadth of research brought to the SCS platform is a strength of the event, as it provides the students with an elaborate overview of the field, and allows them to widen their perspective and learn from each other.

All the delegates rated the presentations and posters. The organizing committee then awarded prizes based on these votes, sponsored by the *Nucleic Acids Research* and *Bioinformatics* journals. The best presentation prize was awarded to Gonzalo Parra for his presentation, “Protein Frustratometer 2: A Tool to Localize Energetic Frustration in Protein Molecules, now with Electrostatics”. The 2nd and 3rd presentation prizes were handed out to Mohammad Oloomi for his work, “The Impact of Sequence Ambiguity on Read Mapping Accuracy”, and to Nathalie Davidson for her presentation, “Differential Expression Analysis for Highly Related Samples”, respectively.

Three poster awards, sponsored by *Bioinformatics*, were handed out to Medhini Narasimhan for her poster, “Predicting Symptom Severity and Contagiousness of Respiratory Viral Infections”, Urszula Czerwinska for her work, “Deconvolution Of Cell and Environment Specific Signals and Their Interactions from Complex Mixtures in Biological Samples” and Mohsen Botlani for his poster, “Quantifying Conformational Ensemble Changes in Proteins Using Inverse Machine Learning”. Thanks to the sponsorship of *F1000*, runner-up awards were presented to Jennie Catlett for her poster, “Modeling and Verification of a Syntrophic Relationship Between Human Gut Microbes”, Libere Ndacayisaba for his poster, “Proteochemometric Machine Learning Models for Predictive Drug Discovery, Target Identification, and Polypharmacology Deconvolution” and Niran Hadad for his work, “Sex-mutual and sexually-dimorphic alterations in hippocampal DNA methylation with aging”.

The generous support from SIB, Elsevier, and Vertex enabled the organizing committee to award 3 travel fellowships to support students in attending the symposium. After a review by the SC community and a final decision by the organizing committee, the travel fellowships were awarded to Mohammad Oloomi, Niran Hadad and Urszula Czerwińska.

SC volunteers also organized a separate career event (called ‘career central’) where 3 established researchers, Prof. Amoolya Singh, Prof. Sarah Teichmann and Prof. Judith Blake gave their insights into the many career paths that are possible for computational biologists. In a discussion panel format, they covered different career related-topics like work-life balance, required expertise and non-academic competences that are necessary for a successful career.

## Fourth European Student Council Symposium in The Hague, The Netherlands

Following the success of the previous ESCS event ^2^, the fourth ESCS was held on September 3, 2016, as a satellite meeting of the 15th ECCB in The Hague, The Netherlands. This year saw the attendance of 60 delegates from 16 countries. The symposium highlighted 3 keynote speakers, 12 student talks, and 36 student poster presentations, spanning diverse topics such as computational drug development, proteomics, chromatin structure prediction, genome sequencing and network analysis.

Among the symposium’s highlights were the inspiring keynotes given by 3 distinguished scientists. The first keynote speaker was Dr. Kris Laukens, a bioinformatics researcher at the Biomedical informatics research center (biomina) and the Advanced Database Research and Modelling (ADReM) group at the University of Antwerp, Belgium. Dr. Laukens discussed the challenges in the field of proteomics and metabolomics analysis, in particular the unambiguous identification of molecules in mass-spectrometry. The second keynote was given by Prof. Roeland Merks, who is a senior researcher at Centrum Wiskunde & Informatica (CWI), the Dutch national researcher for mathematics and computer science in Amsterdam, The Netherlands. Prof. Merks gave us the opportunity to learn about the latest developments of genome scale modelling of the whole microbiome and the challenges of modelling interactions between several bacterial species. The third and final keynote presentation was given by Dr. Núria Lopez-Bigas. She is an ICREA Research professor at the University Pompeu Fabra, and a group leader of the Biomedical Genomics group dedicated to the computational study of cancer at the genomics level. Dr. Lopez-Bigas concluded the day with a career keynote, in which she discussed the choices she has made throughout her career and how these influenced the choices of research areas she has pursued. She reminded the audience of the importance of a good work-life balance and to be perseverant in following one’s own interests in shaping their scientific careers. Besides career advice, Dr. Lopez-Bigas shared her view on the importance of a fitting experimental validation of computational predictions.

The symposium would not have been possible without the generous support from the sponsors The Hyve, Elsevier, The SIB Swiss Institute of Bioinformatics (SIB), and F1000. Importantly, this allowed for travel fellowships to be awarded to three participants, Jonas Ibn-Salem, Lisanna Paladin and Francesco Russo, to attend and present at the symposium. Furthermore, this also allowed us to grant prizes to the best oral and poster presentations of the day. The first oral presentation prize was awarded to Thies Gehrmann for his talk, “Nucleus specific expression in the multi-nuclear mushroom *Agaricus bisporus*”. Sascha Meiers was awarded the second oral presentation prize for his presentation entitled “Functional impact of genomic rearrangements on chromatin organization and transcriptional regulation”. The first poster presentation prize was awarded to Konrad Zych for his work, “Improving potato breeding with computational and functional genomics”. The second poster presentation prize was awarded to Patrick van den Berg on his poster, “An integrated transcriptomics and proteomics study of embryonic stem cell differentiation”.

## Symposia networking activities

Facilitating networking opportunities being a rather major goal of the SC ^3^, there are dedicated events aimed at increasing interactions between attendees at every SC symposium. Ice-breaking events, or “scientific speed dating” as the organization likes to call it, is one such customary occasion at the start of the day. At this meeting launcher, delegates network with a random attendee over a few minutes, before switching to another person. The tradition was carried out this year as well at SCS and ESCS.

Poster sessions at both symposiums also saw lively discussions among attendees. The social event in the evening is yet another opportunity provided by the SC for the delegates to network. This year, Eli Lilly sponsored the event at SCS, which took place at the Dolphin Pool Bar. At ESCS, delegates were escorted to the city center of The Hague for their evening’s social event. In addition to the symposium, the SC also organized the "ice-breaking event" of the overarching ECCB 2016 conference: The Hague@Night Photo Tour.

Throughout ISMB and ECCB this year, the SC booth at the venue served as the hub for student interactions, with future volunteers, job seekers and advertisers, SC alumni and even an occasional principal investigator dropping by.

## Outlook

Twelve years after its first edition in 2005, SCS remains a successful symposium, attracting participants from all over the world. Over the past few years, ESCS has grown to meet similar success, and this year even surpassed SCS in the number of participants and abstract submissions. This is likely due to both better availability of funding as well as the many active Regional Student Groups in this region. Both SCS2016 and ESCS2016 were great successes due to the high quality keynotes, student presentations and posters. Both symposia have paid particular attention to networking opportunities by organizing both an ice-breaking at the start and a social event at the end. The strong social engagement of the SC volunteers and their increasing numbers are clear proofs that this strategy is working.

In 2017, ISMB and ECCB will be jointly organized in Prague, Czech Republic. This meeting will host the 13th Student Council Symposium, and the 5th ESCS will take place in 2018. For information about the Student Council and to learn more about its event, please visit the website: http://www.iscbsc.org.
